# Myeloid Antigen Expression in Childhood Acute Lymphoblastic Leukemia and Its Relevance for Clinical Outcome in Indonesian ALL-2006 Protocol

**DOI:** 10.1155/2012/135186

**Published:** 2012-11-26

**Authors:** Eddy Supriyadi, Anjo J. P. Veerman, Sutaryo, Ignatius Purwanto, Peter M. vd Ven, Jacqueline Cloos

**Affiliations:** ^1^Pediatric Hematology Oncology Division, Department of Pediatrics, Dr. Sardjito Hospital, Faculty of Medicine, Universitas Gadjah Mada, Jl. Kesehatan No. 1, Yogyakarta 55281, Indonesia; ^2^Pediatric Oncology/Hematology Division, Department of Pediatrics, VU University Medical Center, 1007 MB Amsterdam, The Netherlands; ^3^Department of Epidemiology and Biostatistics, VU University Medical Center, 1007 MB Amsterdam, The Netherlands; ^4^Department of Hematology, VU University Medical Center, 1007 MB Amsterdam, The Netherlands

## Abstract

The frequency of acute lymphoblastic leukemia (ALL) patients expressing myeloid antigens on their ALL cells varies between 5 and 36% in several different studies. The clinical relevance of myeloid antigen expression in childhood ALL is controversial. In Indonesian patients, no data were present. Therefore, in Yogyakarta, Indonesia, we analyzed 239 ALL patients who were immunophenotyped including myeloid markers (CD13, CD33, CD117, and/or cMPO). Myeloid antigen expression was found in 25% of patients. Expression of myeloid antigen in B-lineage leukemia was 27%, and in T-lineage leukemia, it was 18% (*P* = 0.15). No association was found between myeloid antigen expression and clinical or biological features. In the whole cohort of patients we did not find a significant association between myeloid antigen expression and survival, although leukemia-free survival at 3 years was higher in the myeloid-negative patients (73% ± 6%) compared to myeloid-positive patients (67% ± 8%). Interestingly, in T-ALL patients, expression of myeloid antigens was an independent adverse prognostic factor (hazard ratio: 3.26, 95% CI: 1.06–9.98, *P* = 0.04). Kaplan-Meier analysis for event-free survival was also significant (log rank *P* = 0.03) in this subgroup. In conclusion, in the Indonesian ALL population, in particular, myeloid antigen-expressing T-ALL patients had a higher chance of having induction failure.

## 1. Introduction

Acute leukemia is a clonal expansion of malignant cells in bone marrow, blood, and other organs. The acute leukemias are classified into acute myeloblastic leukemia (AML) and acute lymphoblastic leukemia (ALL) based on lineage of the blast cells [1–3]. The proliferating lymphoid progenitor cells, arrested in various stages of maturation, can be identified as B-, T-, or mixed-lineage leukemia [4–7]. In childhood ALL, immunophenotyping showed that in B- and T-lineage ALL additional antigens can be expressed that are normally associated with myeloid lineages, that is, CD11b, 13, 14, 15, 33, 36, 65, or 117. In various childhood ALL studies, a large variation is reported on expression of myeloid markers ranging from 5 to 36% of cases [5, 8–15]. Moreover, the clinical relevance of myeloid antigen expression in childhood ALL as a prognostic factor remains controversial [5, 8–12].

Myeloid-positive ALL either originates from progenitor cells, which had not differentiated into either myeloid or lymphoid cells (lineage promiscuity), or from progenitor cells, which have both myeloid and lymphoid features (lineage infidelity) [16, 17]. Some studies showed that children with myeloid-positive ALL had a poorer clinical outcome compared with those of myeloid-negative ALL patients [10, 18, 19]. In contrast, other studies could not confirm this result [5, 8, 11, 12]. The discrepancies between studies may be related to the use of different treatment protocols [20], different populations, as well as different definition criteria of myeloid-positive ALL, which are summarized in Table 1. In this study, the myeloid antigen presenting features of the Indonesian ALL population is determined. In addition, the relevance of myeloid antigen expression on both B-lineage and T-lineage ALL patients for treatment outcome was also analyzed. 

## 2. Materials and Methods

From March 2006 to December 2011, 268 pediatric patients aged <15 years with newly diagnosed ALL were admitted to Dr. Sardjito Hospital, Yogyakarta, Indonesia (DSH). Inclusion criteria for this study are 0–14 completed years of age, no prior treatment, and treated with Indonesia 2006 ALL protocol. Exclusion criteria are prior treatment and mature B-ALL. Among these children, 239 cases fulfilled the inclusion criteria and had complete data available for both clinical parameters and immunophenotyping, including evaluation of myeloid antigen expression. 

### 2.1. Morphologic Diagnosis

Morphologic diagnosis was based on French-American-British (FAB) criteria. Bone marrow (BM) and peripheral blood (PB) smears were stained using May Grünwald-Giemsa, Periodic Acid-Schiff, Sudan Black, and myeloperoxidase. 

### 2.2. Immunophenotyping

BM cells were also tested on 3 colors Becton Dickinson FACSCalibur flowcytometer (BD FACSCalibur) for immunophenotyping. The panel of monoclonal antibodies was CD2, cytoplasmic (c)CD3, CD7 for precursor T-ALL, CD10, CD19, CD22, cCD79a for precursor B-ALL, CD13, CD33, cMPO, and CD117 for myeloid lineage, and CD45, IgG1, IgM, Tdt, and CD34 for non lineage [3, 21]. For membrane staining monoclonal antibodies were added (IgG1, CD2, CD7, CD10, CD13, CD19, CD22, CD33, CD34, CD117, and CD45) to 30 *μ*L of cell suspension in separate polystyrene tubes. This suspension was then incubated in the dark for 15 minutes at room temperature. After incubation, 16.6 *μ*L paraformaldehyde (4%) was added to the cell suspensions and incubated for 4 minutes at room temperature, in the dark. Then, 1 mL Lysing Solution was added and lysed 10 minutes at room temperature, in the dark. Cells were then centrifuged 1500 rpm for 5 minutes, cell pellets was washed twice, and resuspended in 300–500 *μ*L PBS. For Cytoplasmic staining (cIgG1, cCD79a, cCD3, cMPO, and cTdT) cell suspensions were incubated for 15 minutes at room temperature and then washed twice and resuspended 300–500 *μ*L of PBS, run in the BD FACSCalibur. Patients in this study were classified of having myeloid antigen expression (myeloid-positive) if B-lineage or T-lineage ALL cells were expressing one or more of the myeloid antigens CD13, CD33, CD117, and/or cMPO. When a sample was positive for more than one lineage, we used a scoring system adapted from The European Group for the Immunological Classification of Leukemias (EGILs). According to this scoring system, a case is considered biphenotypic when point values are greater than two for the myeloid and one for the lymphoid lineages. 

### 2.3. Treatment Protocol

The Indonesia 2006 ALL Protocol has a 4-drug treatment in induction phase: Corticosteroid (Prednisone/Dexamethasone), Vincristine, L-Asparaginase and Anthracycline. Consolidation phase: Cyclophosphamide, Methotrexate (MTX) intrathecally (i.t.) High Dose MTX, and 6-Mercaptopurine (6-MP). Maintenance phase: 6-MP, MTX, and pulses of Vincristine, Dexamethasone/Prednisone and MTX i.t. 

### 2.4. Statistical Analysis

 Myeloid antigen expression was dichotomized, and the threshold was set at 20%. Association between myeloid antigen expression and clinical and biological variables was tested for using Chi-square test or Fisher's exact test where appropriate. The clinical and biological variables considered were age category, white blood cell count (WBC) at diagnosis, FAB subtype, risk classification, and immunophenotype. Survival curves for event-free survival and leukemia-free survival were computed using Kaplan-Meier analysis, and curves for groups based on myeloid antigen expression were compared with the log-rank test. Survival analyses were performed for the overall group and also separately for the B-ALL and T-ALL group. Hazard ratios for myeloid antigen expression and other variables of interest were computed using Cox regression. Because all patients in high-risk group were given dexamethasone while patients in the standard risk group were randomized for prednisone-dexamethasone, hazard ratios were calculated with correction to the protocol used. Event-free survival (EFS) was calculated from date of start treatment to date of first event: induction failure, death, resistant disease, or relapse. Leukemia-free survival (LFS) was calculated from date of start treatment to date of a leukemic event: induction failure or relapse. No complete remission or induction failure was determined at the end of induction treatment and defined as there were lymphoblasts in peripheral blood or cerebrospinal fluid and/or more than 5% lymphoblasts in the bone marrow. A two-sided *P* value less than 0.05 was used as level for statistical significance. SPSS version 15 was used to analyze the data. Multivariate analyses were restricted to those risk factors that had a *P*-value of less than 0.20 in a univariate analysis. As the variable risk classification was determined on the basis of WBC and age, we excluded this variable from multivariate analyses when both age and WBC were already included in the multivariate analyses. For analysis on the total ALL group, and the B-Lineage ALL group a variable denoting the protocol used was included in all models. 

## 3. Results

Two hundred and sixty-eight patients were immunophenotyped. Twenty-nine patients were excluded (12 patients used Wijaya Kusuma-2000 protocol, 5 had treatment before, 4 refused treatment, 3 patients were inconclusive, 4 got suboptimal treatment, and 1 patient was aged more than 14 years). Two hundred and thirty-nine patients met the inclusion criteria and were analyzed for clinical and biological features. These data are summarized in Table 2. Since one of the national cancer institute criteria for standard risk classification is a child over 1 year old and under 10 years old, age was grouped as 1–9 years and <1 plus >10 years.

We analyzed the immunophenotype of these ALL by a three-color flow cytometer and determined the immunologic classification into B or T-phenotypes. For B-ALL, the most frequent markers were CD19, CD10, cytoplasmic CD79a, and CD22. For T-ALL and the most frequent markers were CD7, cytoplasmic CD3 and CD2. The Immunophenotypic profiles of childhood ALL in Yogyakarta are presented in Table 3.

### 3.1. Overall Patients

**Figure 1:**
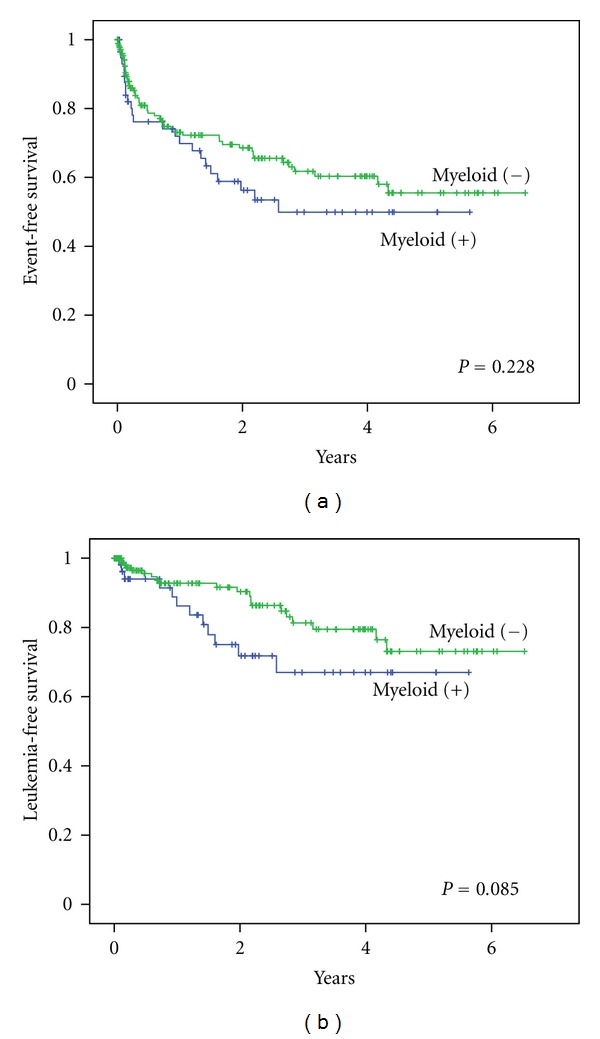
Survival analysis of myeloid expression in overall childhood ALL, treated with Indonesia 2006 protocol. (a) Event-free survival with log rank *P* = 0.228 and (b) Leukemia-free survival with log rank *P* = 0.085.

In the whole patient group, myeloid antigen was expressed in 60 (25%) of 239 patients tested. Majority of patients with morphology ALL-L1 had no myeloid antigen expression (*P* = 0.02). In univariate analysis for EFS, ages at diagnosis and risk group were found to be statistically significant predictors for event-free survival. Multivariate analysis showed that age category was the only factor significant, HR 2.00 (95%CI: 1.23–3.23, *P* = 0.006). Kaplan-Meier analysis of EFS showed no difference for myeloid antigen expression (Figure 1(a)). There was nothing significant in univariate analysis for LFS. Kaplan-Meier analysis showed that LFS at 4 years was 80% ± 5% in the myeloid-negative group compared to 67% ± 8% in the myeloid-positive group (Figure 1(b)). 

The proportion of myeloid antigen expression in the B-lineage group was 27%, while 10 out of 56 patients (18%) were in T-ALL group (*P* = 0.15). Moreover, the groups did not differ in relevant clinical and biological features (Table 4). Since both groups have a distinct clinical prognosis, we also stratified the data for the different lineages.

### 3.2. Precursor B-ALL Patients

Univariate analyses for EFS showed that sex, age at diagnosis, and risk classification were significant factors. On multivariate analysis (correcting for protocol used), nothing remained significant. For LFS, there was no factor significant in univariate analysis. We did not perform multivariate analyses since there were no other variables that had a *P* value smaller than 0.2. 

**Figure 2:**
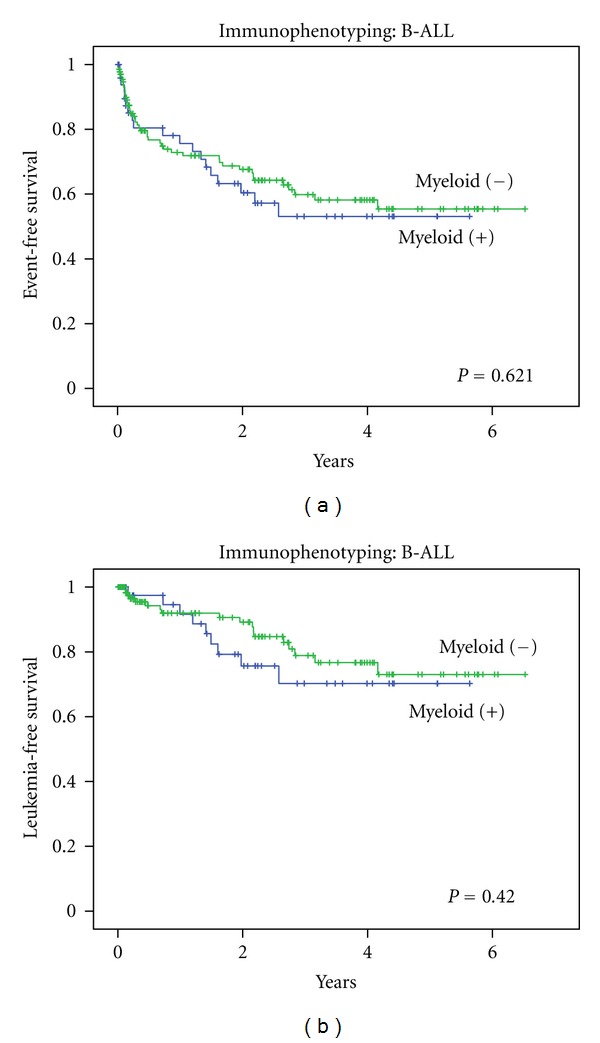
Survival analysis of myeloid expression in B-lineage ALL, treated with Indonesia 2006 protocol. (a) Event-free survival with log rank *P* = 0.621 and (b) Leukemia-free survival with log rank *P* = 0.420.

In the Kaplan-Meier analysis for EFS, precursor B-ALL patients with and without myeloid antigen expression had a similar prognosis. EFS at 4 years for myeloid-positive B-lineage ALL was 53% ± 8%, and for myeloid-negative it was 55% ± 6%, *P* = 0.621 (Figure 2(a)). Kaplan-Meier analysis for LFS also showed no association of survival with myeloid antigen expression (LFS at 4 years was 73% ± 6% for myeloid-negative versus 70% ± 9% for myeloid positive, *P* = 0.420,  Figure 2(b)). 

### 3.3. T-ALL Patients

**Figure 3:**
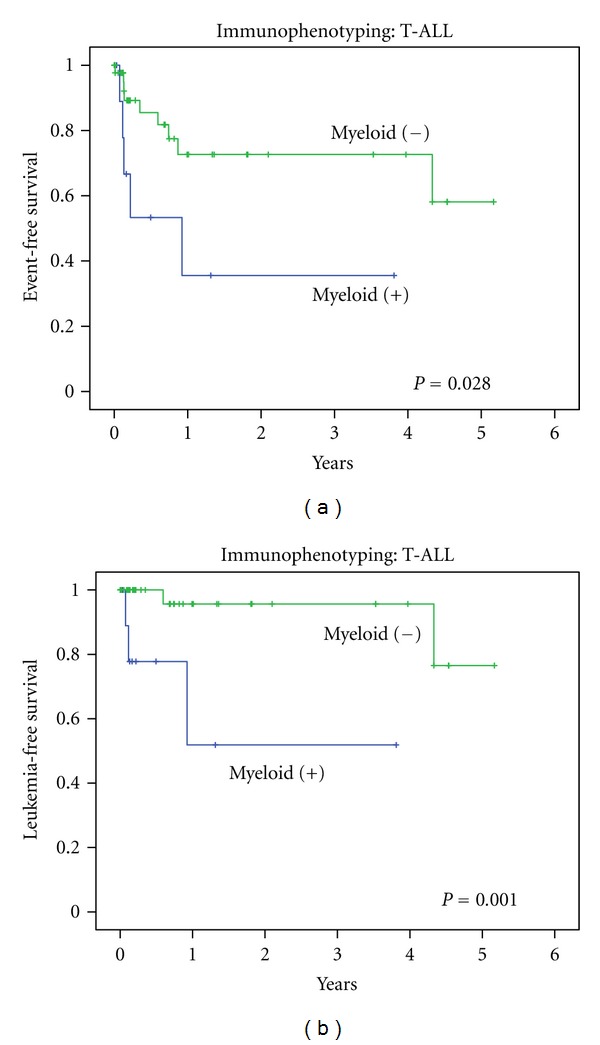
Survival analysis of myeloid expression in T-lineage ALL, treated with Indonesia 2006 protocol. (a) Event free survival with log rank *P* = 0.028 (b) Leukemia free survival with log rank *P* = 0.001.

Univariate analysis for EFS showed no statistically significant parameters except for myeloid antigen expression. Including the known common factors influencing survival, we did perform multivariate analysis and found that expression was an independent risk factor for EFS (HR: 3.26 (1.06–9.98), *P* = 0.04). Kaplan-Meier analysis for EFS (Figure 3(b)) showed that myeloid-positive patients had a worse prognosis. Survival at 4 years for myeloid negative was 58% ± 15% while for myeloid positive was 36% ± 19% (log rank *P* = 0.03). LFS analysis showed that myeloid-positive patients had a worse prognosis. Survival at 4 years for myeloid negative was 77% ± 17% while for myeloid positive was 52% ± 23% (log rank *P* = 0.001). Of the events considered, induction failure was more common in myeloid-positive than in myeloid-negative patients. In myeloid-positive patients, 2 out of 10 patients had this event, while in myeloid-negative patients, no patient had induction failure, Fisher's exact test, *P* = 0.029. 

## 4. Discussion

Leukemic blast cells generally demonstrate lineage fidelity and therefore are believed to reflect stages of normal differentiation of B- or T-lineage [22, 23]. Recent improvement on immunophenotyping makes it possible to detect mixed antigen expression in one cell, including myeloid antigen expression in ALL [10]. The clinical significance of myeloid antigen coexpression has remained controversial [5, 8–15]. We studied the clinical significance of myeloid antigen expression and the treatment outcome in 239 Indonesian children with ALL. The ALL patients analyzed here were classified as having myeloid antigen coexpression according to the expression of at least one of these 4 markers: CD13, CD33, CD117, and cMPO. The 25% incidence of myeloid antigen expression in this Indonesian population was slightly higher compared to what was found in Malaysia by Ng et al. (2000), who reported 23% [15], but lower compared to Den Boer et al. (1999) who found 36% in a European population [9] (Table 1). The variation of those findings may be due to variations in definition; some authors define myeloid positive as two or more myeloid antigens positive, or 1 or more as in this study. In addition, there is variation in the number of monoclonal antibodies used and which varies from only 2 monoclonal antibodies [5, 9, 15] to as many as 6 [8, 11, 12]. Besides these more technical explanations, the myeloid antigen expression may also differ due to ethnical differences.

A study in a single institution conducted by Wiersma et al. (1991) reported a 3-year EFS of 84% for myeloid-negative ALL with WBC < 50.000/mm^3^ compared to 57% for myeloid-positive ALL. For samples with WBC > 50,000/mm^3^, they reported 47% 3-year EFS for myeloid-negative ALL compared to 26% for myeloid-positive ALL. Multivariate analysis showed that myeloid antigen expression was the most important predictor for a poor EFS [10]. This result was similar with the study of Kurec et al. (1991) [18]. In a more recent study, patients with mixed phenotypic acute leukemia (MPAL) especially in B-lineage leukemia with myeloid antigen expression had a lower EFS rate than those with nonmixed acute leukemia [24]. These results were also consistent with the finding that myeloid-negative patients had a higher sensitivity to glucocorticoids than myeloid-positive positive cases, causing a better prognosis in myeloid-negative ALL. This may be related to differences in cellular drug resistance. Leukemic cells from myeloid-positive ALL patients were more resistant to glucocorticoid-induced killing than cells from myeloid-negative ALL patients [25]. 

In our study, only in T-ALL patients, myeloid antigen expression was found to be a significant adverse prognostic factor (*P* = 0.04). It has to be emphasized that only a small number of patients were studied in the T-ALL group. However, LFS analyses also showed a worse prognosis for myeloid-positive patients; LFS at 4 years was 52%, while for myeloid-negative patients, LFS at 3 years was 96% (*P* = 0.001). Although our and many other studies have shown a poor result for myeloid antigen expression in childhood ALL, other investigators found conflicting results. Mirro et al [26] and Pui et al. [11] found that myeloid antigen expression in ALL was not correlated with clinical outcome. Another study conducted by Putti et al., who used six different myeloid antigens, showed that myeloid-positive ALL was not associated with immunophenotype and response to therapy and had no prognostic value [8]. Ng et al. (2000) found that patients with myeloid antigen expression were not significantly different in presenting features and treatment outcome compared to those who had no myeloid antigen expression similar results in B-lineage ALL [15]. It is conceivable that myeloid antigen expression in ALL loses its significance when results of treatment get better, as in many western protocols where EFS reaches levels of 80–90%. 

In B-lineage ALL, there was no association between myeloid antigen expression and treatment outcome. In terms of LFS, however, our results showed that myeloid-negative B-lineage ALL had a slightly better LFS although not statistically significant. In multivariate analysis, myeloid antigen expression was not found prognostically relevant for LFS in B-lineage ALL. In T-lineage ALL, however, cases with myeloid antigen expression had a worse prognosis. In myeloid-negative T-ALL patients (*n* = 46), there was only 1 relapse in the first year of treatment. Hence, therapy for this group should be less intense to prevent toxic deaths.

Factors involved in prognosis are also dependent upon the therapy protocol used. Differences in protocol can modify the effect of prognostic factors. Indeed, theoretically, all prognostic factors lose their effect if the cure rate reaches 100%. In St. Jude, cure rates over 90% are achieved. It is therefore understandable that the prognostic value of any marker, including antigen expression, will be most obvious in situation with lower EFS and LFS, which is the case in Indonesia. In our setting, treatment was more toxic compared to other developing countries but less intensive than in trials in high-income countries. For this reason, our treatments resulted in inadequate control of the disease. For myeloid-positive T-ALL, a more intensive protocol should be implemented, but only in combination with a better supportive care. 

In summary, myeloid antigen expression is common and occurs in 25% of Indonesian children with ALL. In T-ALL patients, myeloid antigen expression was associated with a significantly worse prognosis. It may need more intensive (re)induction protocol. T-ALL patients who did not express myeloid antigens had an excellent leukemia-free survival (only 1 relapse at 3 years and another late relapse). LFS at 3 years was 96%; myeloid-negative T-ALL patients could maybe even do with less intensive treatment.

## Figures and Tables

**Table 1:** Various results of myeloid antigen expression in childhood ALL.

Author, year	No. of cases	Myeloid+ (%)	Myeloid antigens used	Reference
Pui et al., 1990	372	6.4	CD11b, 13, 14, 15, 33, 36	[11]
Pui et al., 1991	410	6.1*	CD11b, 13, 14, 15, 33, 36	[12]
Wiersma et al., 1991	236	23	CD13, 33, 14	[10]
Kurec et al., 1991	51	16	CD13, 14, 15, 33	[18]
Ludwig et al., 1994	736	7	CD13, 33, 65	[13]
Reiter et al., 1994	975	5	CD13, 33, 65	[14]
Uckun et al., 1997	1557	16.7	CD13, 33	[5]
Putti et al., 1998	908	32	CD11b, 13, 14, 15, 33, 65w	[8]
Den Boer et al., 1999	167	36	CD13, 33	[9]
Ng et al., 2000	166	23	CD13, 33	[15]
This study	239	25	CD13, 33, 117, MPO	

*Myeloid+ ALL only if the leukemic cells coexpressed two or more myeloid-associated antigens; Myeloid+: myeloid-associated antigen expression; CD: cluster of differentiation; cMPO: cytoplasmic myeloperoxidase.

**Table 2:** Myeloid antigen expression in childhood acute lymphoblastic leukemia in Yogyakarta, Indonesia.

Variable	Patients (%) *n* = 239	Myeloid positive *n* (%)	Myeloid negative *n* (%)	*P* value
		60 (25)	179 (75)	
Sex				
Male	139 (58)	37 (27)	102 (73)	0.52
Female	100 (42)	23 (23)	77 (77)	
Age				
1–9 years	188 (79)	49 (26)	139 (74)	0.51
<1 & 10–14 yrs	51 (21)	11 (22)	40 (78)	
White blood cell count				
<50.000/mm^3^	185 (77)	47 (25)	138 (75)	0.84
>50.000/mm^3^	54 (23)	13 (24)	41 (76)	
French-American-British classification				
ALL-L1	198 (83)	44 (22)	154 (78)	**0.02**
ALL-L2	41 (17)	16 (39)	25 (61)	
Immunophenotyping				
B-lineage	183 (77)	50 (27)	133 (73)	0.15
T-lineage	56 (23)	10 (18)	46 (82)	
Risk classification (NCI based)				
Standard risk	95 (40)	29 (31)	66 (69)	0.17
High risk	144 (60)	31 (22)	113 (79)	

**Table 3:** Immunophenotypic profiles of childhood ALL in Yogyakarta, Indonesia.

Marker	B-lineage	T-Lineage
	%	%
CD2	5	76
CD3	25	80
CD7	8	91
CD10	82	35
CD19	93	14
CD22	71	9
CD79a	80	10
CD34	56	22
TdT	62	43
CD33	10	12
CD13	21	22
CD117	4	7
MPO	0	4

**Table 4:** Myeloid expression in B-lineage and T-lineage ALL in Yogyakarta, Indonesia.

Variable	B-lineage ALL, *n* = 183			T-Lineage ALL, *n* = 56		
	*n* (%)	My+ *n* (%)	*P*	*n* (%)	My+ *n* (%)	*P*
Sex						
Male	105 (57)	30 (27)	0.66	34 (61)	7 (21)	0.51
Female	78 (43)	20 (26)		22 (39)	3 (14)	
Age						
1–9 years	145 (80)	41 (28)	0.57	43 (79)	8 (19)	0.79
<1 & 10–14 yrs	38 (20)	9 (24)		13 (21)	2 (15)	
White blood cell count						
<50.000/mm^3^	149 (81)	41 (28)	0.90	36 (64)	6 (17)	0.76
>50.000/mm^3^	34 (19)	9 (27)		20 (36)	4 (20)	
French-American-British classification						
ALL-L1	149 (82)	37 (25)	0.11	49 (88)	7 (14)	0.07
ALL-L2	34 (18)	13 (38)		7 (12)	3 (43)	
Risk classification (NCI based)						
Standard risk	95 (52)	29 (31)	0.31	0	0	—
High risk	88 (48)	21 (24)		56 (100)	10 (18)	
